# Paradigm Shift in Radiation Biology/Radiation Oncology—Exploitation of the “H_2_O_2_ Effect” for Radiotherapy Using Low-LET (Linear Energy Transfer) Radiation such as X-rays and High-Energy Electrons

**DOI:** 10.3390/cancers8030028

**Published:** 2016-02-25

**Authors:** Yasuhiro Ogawa

**Affiliations:** Hyogo Prefectural Kakogawa Medical Center, Kakogawa, Hyogo 675-8555, Japan; ogaway@kochi-u.ac.jp; Tel.: +81-79-497-7001; Fax: +81-79-438-3757

**Keywords:** hydrogen peroxide, radiosensitizer, sodium hyaluronate, radiotherapy, KORTUC, radiation therapy, H_2_O_2_ effect, tumor hypoxia

## Abstract

Most radiation biologists/radiation oncologists have long accepted the concept that the biologic effects of radiation principally involve damage to deoxyribonucleic acid (DNA), which is the critical target, as described in “Radiobiology for the Radiologist”, by E.J. Hall and A.J. Giaccia [1]. Although the concepts of direct and indirect effects of radiation are fully applicable to low-LET (linear energy transfer) radioresistant tumor cells/normal tissues such as osteosarcoma cells and chondrocytes, it is believed that radiation-associated damage to DNA does not play a major role in the mechanism of cell death in low-LET radiosensitive tumors/normal tissues such as malignant lymphoma cells and lymphocytes. Hall and Giaccia describe lymphocytes as very radiosensitive, based largely on apoptosis subsequent to irradiation. As described in this review, apoptosis of lymphocytes and lymphoma cells is actually induced by the “hydrogen peroxide (H_2_O_2_) effect”, which I propose in this review article for the first time. The mechanism of lymphocyte death via the H_2_O_2_ effect represents an ideal model to develop the enhancement method of radiosensitivity for radiation therapy of malignant neoplasms. In terms of imitating the high radiosensitivity of lymphocytes, osteosarcoma cells (representative of low-LET radioresistant cells) might be the ideal model for indicating the conversion of cells from radioresistant to radiosensitive utilizing the H_2_O_2_ effect. External beam radiation such as X-rays and high-energy electrons for use in modern radiotherapy are generally produced using a linear accelerator. We theorized that when tumors are irradiated in the presence of H_2_O_2_, the activities of anti-oxidative enzymes such as peroxidases and catalase are blocked and oxygen molecules are produced at the same time via the H_2_O_2_ effect, resulting in oxidative damage to low-LET radioresistant tumor cells, thereby rendering them highly sensitive to irradiation. In this review, this potential paradigm shift in modern radiation biology/radiation oncology is discussed in detail in terms of overcoming drug/radiation resistance in radiation therapy and/or anti-cancer chemotherapy.

## 1. Introduction

Considering the importance of fundamental radiation interactions in modern radiation biology, there is significantly more emphasis placed on reporting outcomes from radiation oncology across the scientific field. This review focuses on a potentially paradigm-shifting novel technique for rendering radioresistant tumor cells sensitive to low-LET (linear energy transfer) radiation such as X-rays and high-energy electrons commonly used in modern radiation oncology. What are the most important considerations in terms of the interaction of living cells and tissues with radiation? In clinical radiation oncology, this would include damage to both malignant neoplasms (cancer) and normal organs/tissues surrounding the lesion. Accordingly, it is critically important to determine how different kinds of radiation interact specifically with cancerous and/or normal tissues, and how we can most appropriately utilize different types of radiation to improve the therapeutic effects of radiation therapy.

## 2. Beam Radiation Therapy

The Linear accelerator (Linac) was first used to treat a patient in 1952. Since then, understanding of the physical aspects of radiation therapy has progressed significantly. Heavy-particle and proton beam therapies have recently become available clinically. Intensity-modulated radiation therapy (IMRT) and stereotactic radiation therapy (SRT) using high-quality Linac radiotherapy systems are now utilized worldwide. Although carbon beam therapy is somewhat promising because the therapeutic effect is thought to be unaffected by tumor hypoxia, it has several important limitations. For example, the costs associated with constructing carbon beam facilities are enormous, and many radiation oncologists are inexperienced with use of the characteristic Bragg’s peak in the therapy of superficial tumors such as breast cancer.

## 3. Interaction of Low-LET (Linear Energy Transfer) Radiation with Cancerous and Normal Tissues

X-rays and electrons produced by Linacs are widely used clinically and thus play a primary role in radiation therapy. Both X-rays and electrons are forms of low-LET radiation. How low-LET radiation interacts with malignant neoplasms and normal organs/tissues is a critical consideration.

Irradiation has direct effects on deoxyribonucleic acid (DNA). For example, a secondary electron resulting from absorption of an X-ray photon interacts with the DNA to produce an effect, and this is the dominant process associated with high-LET radiation such as carbon ion beams [1].

Irradiation may also exert indirect effects. Secondary electrons can interact with a water molecule, for example, to produce a hydroxyl radical, which in turn damages the DNA molecule [1]. The DNA helix has a diameter of about 2 nm. It is estimated that free radicals produced in a cylinder with a diameter double that of the DNA helix can affect the DNA. Indirect action is the dominant form of interaction between biological material and less-concentrated ionizing radiation sources, such as X-rays and electrons produced by a Linac.

Water comprises approximately two thirds of human body; therefore, approximately two-thirds of radiation delivered during therapy affects water molecules. The subsequent degradation of water molecules produces hydroxyl radicals, which can then oxidize other molecules or result in H_2_O_2_, thus affecting biological processes. Without oxygen molecules, radiation would have no indirect effects.

The oxygen enhancement ratio (OER) is defined as the “iso-effective dose without oxygen molecules” divided by the “iso-effective dose under oxygen supply”. For X-rays and electrons, the OER is estimated at 2.5~3.0 [1], indicating that in the absence of oxygen molecules, the therapeutic effect of X-rays and electrons is reduced to approximately one-third of the effect that would be expected if oxygen molecules were present in cancerous tissues. Therefore, relatively large tumors over several centimeters in diameter generally cannot be destroyed by radiation therapy in routine clinical practice. As such, “locally curing such a big tumor” is considered the most important issue in therapy using Linac-produced radiation.

Although progress in radiation biology has not kept pace with advances in radiation physics in terms of improvements in physical and spatial dose distributions in IMRT and SRT, it is thought that radiation biology will ultimately be more helpful in clinical radiation therapy. To achieve that, it will be essential to identify the most important factors involved in the interaction between X-rays/electrons and malignant neoplasms (cancers) and to promote fundamental/clinical research to fully characterize how these factors affect therapeutic applications. 

## 4. Significance of Oxygen Molecules in the Interaction between Radiation and Cancers

Most radiation therapy is currently performed using X-rays or high-energy electron beams produced by a Linac; however, these forms of radiation are not ideal in terms of their low-LET characteristics. Almost two-thirds of the radiation-induced damage to cancer cells caused by low-LET radiation results from the production of reactive oxygen species (ROS) in irradiated cells [1]. Therefore, oxygen is essential for fixation of the radicals produced by irradiation.

## 5. History of the Development of Methods to Overcome Tumor Hypoxia and the Need to Reconsider Current Approaches

Over the past 40 years, various radiosensitizers (e.g., metronidazole, misonidazole, etanidazole, and nimorazole) have been developed to increase radiotherapeutic effects [2,3,4]. However, aside from nimorazole, which is approved for use in clinical practice in Denmark, these agents have not been approved for clinical use elsewhere because their effects have not been fully characterized and they exhibit various potential side effects, such as peripheral neuropathy.

For more than four decades, enormous effort has been expended in an attempt to overcome the problem of tumor hypoxia. Although dozens of clinical trials have been performed, the results of most have been inconclusive or of borderline significance. Overgaard and colleagues performed a meta-analysis of these trials [5]. They identified 10,602 patients treated in 82 randomized clinical trials involving hyperbaric oxygen, radiosensitizers, carbogen breathing, or blood transfusions. Tumor sites included the bladder, uterine cervix, central nervous system, head and neck, and lung.

Overall, local tumor control was improved by 4.6%, survival by 2.8%, and the complication rate increased by only 0.6%, which was not statistically significant. The largest number of trials involved head and neck tumors, which also showed the greatest benefit. It was concluded that hypoxia might be only marginally problematic in most adenocarcinomas but highly important in squamous cell carcinomas.

Unfortunately, the above-mentioned procedures for overcoming tumor hypoxia have not proven to be of sufficient clinical benefit. Therefore, besides oxygen molecules, we also need to focus on ways to block the mechanisms cancer cells use to defend against oxidative stress associated with radiation therapy and/or anti-cancer chemotherapy. Anti-oxidative enzymes such as peroxidases and catalase constitute the most important impediments to oxidation-based therapies.

## 6. Do the Therapeutic Effects of X-rays and Electrons Produced by a Linac Involve DNA Damage?

**Figure 1:**
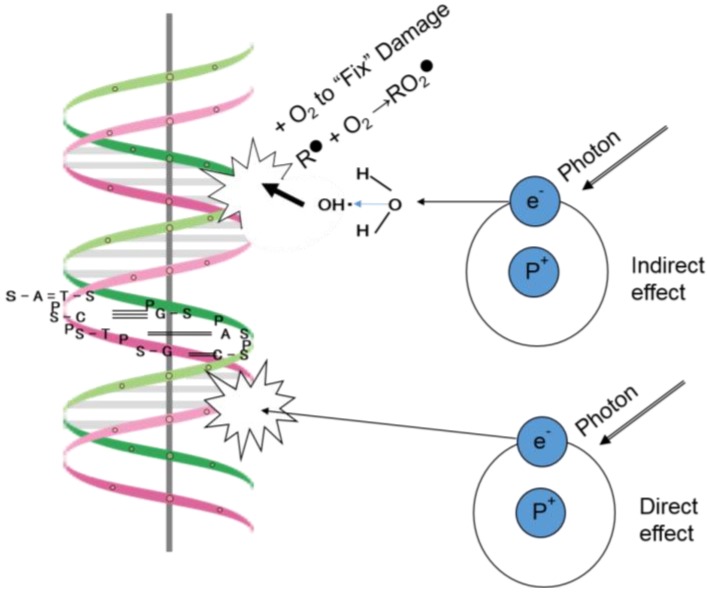
Direct and indirect actions of radiation (adapted from Figure 1.8 in Radiobiology for the Radiologist by E.J. Hall and A.J. Giaccia [1]).

The biologic effects of radiation are thought to result principally from damage to DNA, which is the critical target of radiotherapy [1]. The detailed description (Figure 1) of the direct and indirect actions of radiation by Hall and Giaccia has influenced almost every radiation biologist/radiation oncologist since their work was published. With respect to the direct action of radiation, a secondary electron resulting from absorption of an X-ray photon interacts with the DNA molecule to produce an effect, and this is the dominant process associated with high LET radiation sources such as carbon ion beams. With respect to the indirect action of radiation, the secondary electron may interact with a water molecule to producing a hydroxyl radical that in turn damages to the DNA molecule.

The DNA helix has a diameter of about 2 nm. It is estimated that free radicals produced in a cylinder with a diameter double that of the DNA helix can affect a DNA molecule. Less concentrated forms of ionizing radiation, such as X-rays and electrons produced by a Linac, predominantly involve indirect action [1].

The number of free radicals produced in a cylinder with a diameter (about 2 nm) double that of the DNA helix is thought to be much lower than the number produced in the cytoplasm of cells irradiated with X-rays. Therefore, Hall and Giaccia's description [1] of the indirect action of radiation is only applicable for low-LET radioresistant cancer cells, which exhibit considerable anti-oxidative activity in terms of radical scavenging. It is therefore necessary to reconsider the effects resulting from the interaction between X-rays and water molecules, which comprise two-thirds of the volume of the human body, including cancerous and normal tissues.

## 7. Anti-Oxidative Enzymes in the Therapeutic Effects of X-rays and Electrons Produced by Linac

Anti-oxidative enzymes such as peroxidases and catalase scavenge radicals produced by low-LET irradiation sources, diminishing the therapeutic effects of irradiation. Peroxidases and catalase are abundant in most tumor tissues, as demonstrated by the need to block endogenous peroxidase activity before immunohistochemical staining of fresh tumor tissues [6,7]. Examples of tumors that are resistant to low-LET radiation include those that have many hypoxic tumor cells and/or an abundance of anti-oxidative enzymes. Low-LET radioresistant tumor cells are thought to reside primarily in hypoxic regions of the tumor. 

## 8. Possible Mechanism of Radiosensitivity Augmentation—Special Reference to “Peroxidase Blockade” as a Model of Radiosensitization

When pre-treating cancer tissues for immunohistochemical staining, it is usually necessary to block the activity of peroxidases in order to avoid non-specific staining [6,7]. Most cancer tissues contain an abundance of peroxidases and catalase. However, these anti-oxidative enzymes can be easily inactivated by low concentrations of hydrogen peroxide (H_2_O_2_; approximately 0.3% *w*/*v*), which produces microbubbles of oxygen upon application to the tissues. Pre-treatment with H_2_O_2_ inactivates peroxidases and catalase, resulting in negative staining for peroxidase activity in malignant neoplasms. This suggests that H_2_O_2_ may be clinically useful in radiotherapy in terms of providing an ideal microenvironment in radiotherapy using low-LET radiation such as Linac-produced X-rays and electrons. As tumors in this state of peroxide blockade and hyperoxia are considered ideal for low-LET radiotherapy in terms of the presence of abundant oxygen and the inactivation of anti-oxidative enzymes, it is considered critically important to reproduce these conditions for targeting cancer tissues when performing clinical radiotherapy using low-LET radiation such as X-rays and electrons produced by currently available Linac instruments. Though, recent studies also described that H_2_O_2_ production and high ROS stress within tumor microenvironment play key roles in immune dysfunction and metabolic shift [8,9]. Therefore, an appropriate H_2_O_2_ application and its precise targeting are considered essential in a clinical use.

## 9. Mechanism of Radiation-Induced Apoptosis of Lymphocytes

Among the numerous types of human cells, peripheral T cells are considered to be representative in terms of their high level of radiosensitivity [10,11]. Hall and Giaccia [1] describe lymphocytes as very radiosensitive, largely because of apoptosis. B cells are more radiosensitive than T cells, and overall, their radiosensitivity, as measured by clonogenic assay, is similar to that of hematopoietic stem cells. Because human peripheral lymphocytes and lymphoma/leukemia cells of lymphoid tissue origin lack peroxidase activity, these cells are quite susceptible to oxidative stress such as that caused by ROS, including H_2_O_2_ [12,13].

**Figure 2:**
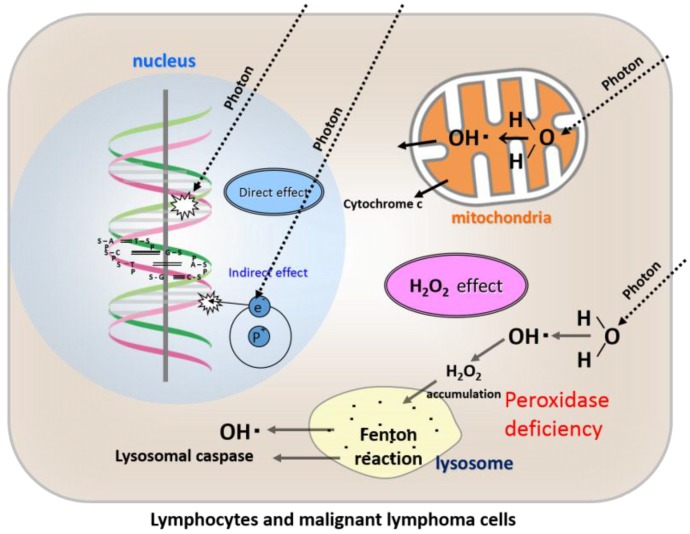
Direct effects, indirect effects, and our newly proposed “H_2_O_2_ effect” of radiation in lymphocytes and malignant lymphoma cells.

When T cells are irradiated, H_2_O_2_ produced by ROS accumulates in the cytoplasm of lymphocytes and then moves into the lysosomes, where it causes lysosomal membrane dysfunction and ultimately apoptosis [14,15] (Figure 2). Damage to DNA resulting from irradiation is not considered to play a major role in the mechanism of lymphocyte death. Instead of damage to DNA, the “H_2_O_2_ effect” is proposed as a mechanism playing a major role in this respect. Moreover, it is thought that these mechanisms are considered to explain the high rediosensitivity of lymphocytes and malignant lymphomas. Therefore, in terms of imitating the high radiosensitivity of lymphocytes and malignant lymphomas, osteosarcoma cells (which are representatives of low-LET radioresistant tumor cells) can be an ideal model of the conversion of the cells from radioresistant to radiosensitive, by exploiting the “H_2_O_2_ effect” in terms of peroxidase blockade and hyperoxia.

As lymphocytes lack peroxidase activity, H_2_O_2_ produced from ROS following irradiation accumulates in the cytoplasm and then moves into the lysosomes, resulting in lysosomal membrane dysfunction and ultimately apoptosis.

## 10. The Low-LET Radioresistant Osteosarcoma Cell Line, HS-Os-1

Osteosarcoma cells are highly resistant to low-LET radiation, and we demonstrated by peroxidase staining that these cells contain an abundance of peroxidases [14]. By adding low concentrations of H_2_O_2_ to the culture medium, osteosarcoma cells were easily converted to a highly radiosensitive state [15]. Based on studies using low-LET radioresistant HS-Os-1 osteosarcoma cells and PC-3 prostate cancer cells, we found that sensitivity to X-ray irradiation could be easily increased and apoptosis rapidly induced in the presence of low concentrations of H_2_O_2_ in the culture medium during irradiation [16,17,18,19]. In our previous study, we showed that radioresistant osteosarcoma cells and chondrocytes exhibit minimal ROS formation, even when exposed to a relatively high radiation dose of 30 Gy as a single fraction [17,20]. In contrast, when irradiation was performed in the presence of a low concentration of H_2_O_2_ (0.1 mM), abundant ROS were produced in the cells, leading to apoptosis, despite exposure to a relatively low radiation dose of 10 Gy as a single fraction [17].

**Figure 3:**
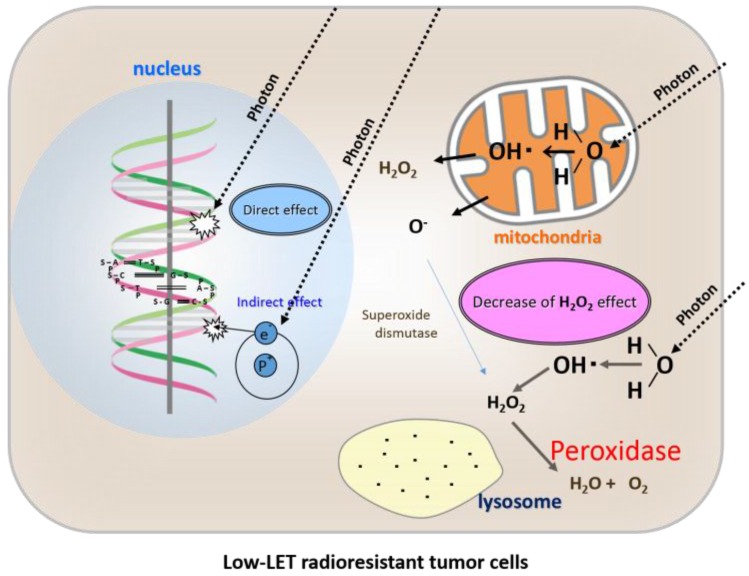
Direct effects, indirect effects, and a shortage of the “H_2_O_2_ effect” of radiation therapy in low-LET radioresistant tumor cells.

**Figure 4:**
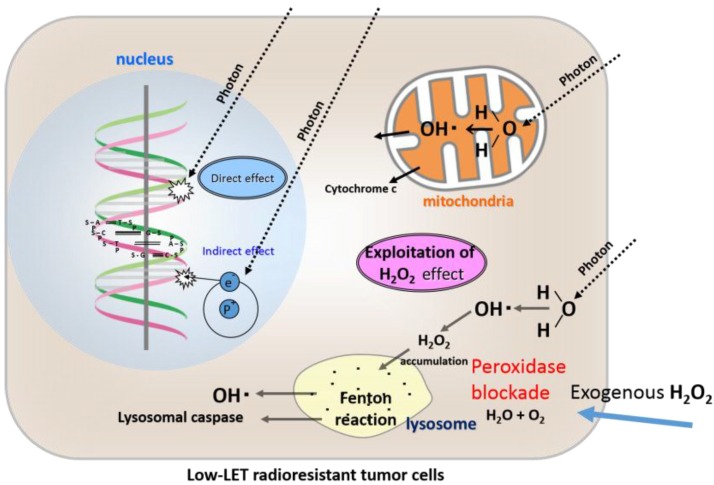
Direct effects, indirect effects, and an exploitation of our newly proposed “H_2_O_2_ effect” by an exogenous H_2_O_2_ supply for low-LET radiation in chondrocytes and osteosarcoma cells in terms of blockade of high peroxidase activity and hyperoxia.

Based on these data, we concluded that osteosarcoma cells are resistant to X-ray irradiation because of high peroxidases and catalase activity [16] (Figure 3). Therefore, by providing exogenous H_2_O_2_ before irradiation, the activities of anti-oxidative enzymes can be blocked and oxygen molecules can be produced at the same time, leading to oxidative damage to low-LET radioresistant tumor cells. By such a mechanism, low-LET radioresistant tumor cells can be converted into highly radiosensitive cells [19,20] (Figure 4).

Osteosarcoma cells are characterized by high peroxidases and catalase activity, which degrades H_2_O_2_ into water and oxygen, and prevents its accumulation in lysosomes. Therefore, lysosomal membrane dysfunction cannot be induced by low-LET irradiation.

Osteosarcoma cells are characterized by high peroxidases and catalase activity, which are inactivated by exogenous H_2_O_2_ supply, and excess H_2_O_2_ accumulates in the cytoplasm and then moves into the lysosomes, resulting in lysosomal membrane dysfunction and ultimately apoptosis following low-LET irradiation.

## 11. What Is KORTUC I: Kochi Oxydol-Radiation Therapy for Unresectable Carcinomas, Type I?

In a previous study, we developed KORTUC I (Kochi Oxydol-Radiation Therapy for Unresectable Carcinomas, Type I), a radiosensitization method for the body surface that markedly enhances the radiosensitivity of various types of superficially exposed and locally advanced neoplasms [21]. In KORTUC I, sterilized cotton soaked in a 3% *w*/*v* H_2_O_2_ solution (Oxydol) is applied to the cancer as a bolus and then irradiated with a 15-MeV electron beam from a Linac. Remarkable therapeutic effects were achieved in our study using this technique, with complete disappearance of the tumor.

## 12. Intra-Tumoral Injection of H_2_O_2_

Meningiomas can be treated using intratumoral injection of 3–5 mL of Oxydol (3% *w*/*v* H_2_O_2_) followed by surgery, with no significant side effects [20]. In KORTUC II, only 3–6 mL of diluted Oxydol (0.5% *w*/*v* H_2_O_2_) mixed with sodium hyaluronate is used for intra-tumoral injection, which represents a much lower dose than that used in the method described in the article [22].

After much experimentation, we found that irritation of the skin, mucous membranes, and other tissues in the presence of H_2_O_2_ can be reduced by combining sodium hyaluronate with H_2_O_2_ [23].

## 13. Confirmation of the Safety of Intra-Tumoral Injection of H_2_O_2_ with Various Viscosity-Increasing Agents Using a Mouse Tumor Model

To increase the viscosity of the H_2_O_2_ and delay degradation of H_2_O_2_ in human tumor tissue, we examined the effect of mixing various agents with H_2_O_2_ using a mouse tumor model. The results of these experiments indicated that sodium hyaluronate is the most appropriate agent for combined use with H_2_O_2_ [24].

## 14. Development of the KORTUC II Radiosensitization Method

**Figure 5:**
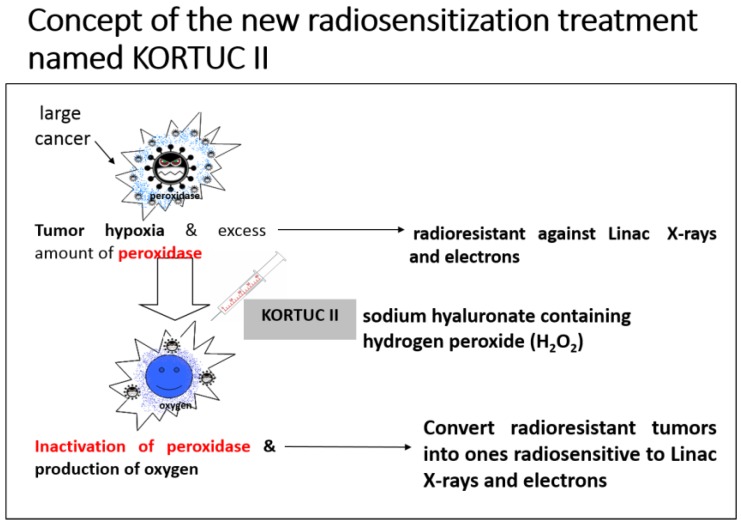
Concept of the novel KORTUC II (Kochi Oxydol-Radiation Therapy for Unresectable Carcinomas, Type II) radiosensitization method.

Based on our clinical experience using KORTUC I, and the experimental results described above, we developed the KORTUC II radiosensitization method for intra-tumoral injection of H_2_O_2_ and sodium hyaluronate for the treatment of various tumors that are not superficially exposed [25]. The concepts underlying this new enzyme-targeting radiosensitization treatment are shown in Figure 5. The aim was to formulate an effective radiosensitizer that causes minimal irritation, can be safely injected into human tumors, and preserves the oxygen concentration in the tumor tissue for more than 24 h.

When treating low-LET radioresistant tumors, it is necessary to both inactivate anti-oxidative enzymes and produce oxygen in the tumor tissue to augment the effects of X-rays and high-energy electron beams. The combined use of sodium hyaluronate and H_2_O_2_ preserves the oxygen concentration in the tumor tissue for more than 24 h. Recently, the injection of H_2_O_2_ into pelvic soft tissue between the rectum and urinary bladder was reported to be safe for use in radiation therapy for prostate cancer, in terms of avoiding late side effects resulting from an overdose of radiation to the rectum wall [26].

Application of the KORTUC II method produces a superior radiotherapeutic effect using low-LET beams. This is true even for low-LET radioresistant tumors containing many hypoxic tumor cells and an abundance of peroxidases and catalase.

KORTUC II is a totally new enzyme-targeting radiosensitization treatment modality developed at Kochi University, Japan, and it is the first reported radiosensitization method for intratumoral injection of non-superficially exposed tumors [25,27]. KORTUC II can be used with conventional Linac instruments, of which there are currently more than 1000 in Japan. In KORTUC II, a mixed solution of H_2_O_2_ and sodium hyaluronate inactivates the anti-oxidative enzyme peroxidase in the tumor tissue. In this reaction, oxygen is produced by the degradation of H_2_O_2_, partial oxygen pressure is increased, and radicals that are produced in tumor cells by X-ray irradiation are oxidized. Moreover, with the decline in peroxidase activity in tumor cells, H_2_O_2_, which is the last product of radicals and ROS, cannot be degraded and thus accumulates in the cell. As a result of this “H_2_O_2_ effect”, mitochondrial and lysosomal apoptosis of tumor cells is easily induced following low-LET irradiation [16,18,20]. Through the above-mentioned mechanism, KORTUC II facilitates the conversion of low-LET radioresistant cells into radiosensitive cells [17,19]. The method is now considered essential for enhancing the therapeutic effect of radiotherapy using Linac-produced X-rays and electrons.

KORTUC II is a very safe method in terms of the application of H_2_O_2_ and sodium hyaluronate to body tissues in order to augment the therapeutic effect of radiotherapy and/or chemotherapy. Both H_2_O_2_ and sodium hyaluronate are present naturally in the human body tissues; H_2_O_2_ is present in saliva, and sodium hyaluronate is found in subdermal tissues. In fact, it has been nearly 50 years since arterial injection of 250 mL of 0.12% *w*/*v* H_2_O_2_ was first performed for radiotherapy of patients with head and neck cancers, in 1967 [28]. Furthermore, KORTUC II is an enzyme-targeting therapy that inactivates the anti-oxidative enzymes peroxidases and catalase, which are the primary anti-oxidative enzymes in the human body. Therefore, KORTUC II targets an essential defense system of tumor cells.

With respect to intra-tumoral injection of the KORTUC II agent under ultrasonographic or CT guidance, the Bragg's peak of a heavy particle beam, such as a carbon beam, can be captured by imaging-guided injection of the radiosensitizer in terms of local radiosensitization limited to the targeting tumor and tissues around the tumor. A major characteristics of KORTUC II is the use of sodium hyaluronate to increase viscosity in terms of preserving the partial oxygen pressure in the tumor tissue.

Since the report almost 50 years ago of intra-arterial injection of H_2_O_2_ for tumor radiosensitization [28], there have been no further related publications. The reason for this may be that the agent could not be precisely injected into the tumor tissue because ultrasonographic equipment and/or CT were not widely available. Moreover, intra-arterial injection of an excess amount of H_2_O_2_ can result in oxygen embolism from oxygen produced by the degradation of H_2_O_2_ by peroxidases in red and white blood cells. For this reason, injecting the KORTUC II agent into the great vessels is contraindicated. Therefore, the agent should be injected intra-tumorally under imaging guidance. Intra-tumoral injection of the KORTUC II agent is now possible thanks to the development of modern diagnostic imaging techniques such as power Doppler ultrasonography and MDCT (multi-row detector CT).

The most important consideration in using KORTUC II is that the agent be injected into the tumor with a homogeneous distribution using a relatively fine needle (e.g., 23-G) under imaging guidance, mainly ultrasonographic. The key characteristic of this treatment is that every radioresistant tumor in the area in which the agent is homogenously injected is converted to a radiosensitive tumor. Hence, KORTUC II is a novel imaging-guided, enzyme-targeting radiosensitization method.

KORTUC II is safe and effective and considerably less expensive than other methods; therefore, it has great potential to become a viable non-invasive alternative to surgical procedures for the treatment of most of low-LET radioresistant neoplasms. Non-surgical BCT (breast-conserving treatment) can be performed using KORTUC II [27,29]. The method has three major characteristics: imaging guidance by ultrasonography; targeting of peroxidases and catalase; and targeting of breast cancer stem cells via the CD44. Local control and cosmesis have remained excellent at the most recent follow-up, with acceptable rates of acute/late toxicity. Non-surgical BCT (KORTUC-BCT) can be performed safely and effectively for patients with stage I or II breast cancer [29,30,31,32]. To demonstrate this clearly, it will be essential to conduct randomized clinical trials in Western countries, as well as in Japan. The KORTUC II agent has been patented in many leading countries, including Japan, the UK, Germany, France, Australia, Canada, and China.

### Formulation Example

A syringe containing a hyaluronic acid preparation (2.5 mL) with a 1% *w*/*v* concentration of sodium hyaluronate (ARTZ Dispo, Seikagaku Corporation, Tokyo, Japan) is used. This preparation contains 25 mg of sodium hyaluronate, 2.5 mg of l-methionine, sodium chloride, potassium phosphate, crystalline sodium dihydrogen phosphate, and an isotonizing agent. The preparation is a colorless, transparent, and viscous, aqueous solution having with a pH of 6.8 to 7.8, specific osmotic pressure of 1.0 to 1.2 (relative to physiological saline), and a weight-average molecular weight of 600,000 to 1.2 million. To this, 0.5 mL of a 3% *w*/*v* solution of H_2_O_2_ (Oxydol, Ken-ei Pharmaceutical Co. Ltd., Osaka, Japan) is added immediately before use and mixed well. The H_2_O_2_ is aseptically prepared and provided as a small vial containing 0.5 mL of 3% *w*/*v* H_2_O_2_ by the Department of Pharmacy, Kochi University Hospital. The sensitizer used in our study has a sodium hyaluronate concentration of 0.83% *w*/*v* and a H_2_O_2_ concentration of approximately 0.5% *w*/*v*.

For eliminating local pain due to injection of the KORTUC agent, in March 2008 we began including approximately 0.5 mL of 1% *w*/*v* lidocaine with the KORTUC injection. Since that time, none of the patients treated using KORTUC II have complained of significant local pain.

## 15. Conclusions

Because the KORTUC II radiosensitization method is safe and considerably less expensive than other methods and is suitable for the treatment of almost every type of low-LET radioresistant neoplasm, it has the potential for immediate worldwide use.

Utilizing the KORTUC II radiosensitization method based on exploiting our newly proposed mechanism of the “H_2_O_2_ effect” in low-LET radiation therapy, our goal is to establish and widely promote non-surgical chemo-radiosensitization treatment for early stages breast cancer (KORTUC-BCT) [27,29,30,31,32] and locally advanced breast cancer (KORTUC-LABC) [33]; electron-radiosensitization for lesions with local recurrence (KORTUC-REC), including post-radiotherapy lesions; transcatheter arterial chemo-sensitizing embolization treatment (KORTUC-TACE) for locally advanced hepatocellular carcinoma; and intra-operative radiosensitization treatment for locally-advanced stage IVa pancreatic carcinoma (KORTUC-IOR) [34]. We are also trying to improve for developing a new long-acting radiosensitizer for once-weekly intratumoral injection named New KORTUC [35].

## References

[1] Hall E.J., Giaccia A.J. (2012). Physics and chemistry of radiation absorption. Radiobiology for the Radiologist.

[2] Jette D.C., Wiebe L.I., Chapman J.D. (1983). Synthesis and *in vivo* studies of the radiosensitizer 4-[82Br]bromomisonidazole. Int. J. Nuclear Med..

[3] Coleman C.N. (1985). Hypoxic cell radiosensitizers: Expectations and progress in drug development. Int. J. Radiat. Oncol. Biol. Phys..

[4] Overgaard J. (1994). Clinical evaluation of nitroimidazoles as modifiers of hypoxia in solid tumors. Oncol. Res..

[5] Overgaard J. (2007). Hypoxic radiosensitization: Adored and ignored. J. Clin. Oncol..

[6] Ogawa Y., Nishioka A., Hamada N., Terashima M., Inomata T., Yoshida S., Seguchi H., Kishimoto S. (1997). Immunohistochemical study of c-fos-positive lymphocytes infiltrated into human squamous cell carcinomas of the head and neck during radiation therapy and its clinical significance. Clin. Cancer Res..

[7] Ogawa Y., Nishioka A., Hamada N., Terashima M., Inomata T., Yoshida S., Seguchi H., Kishimoto S. (1997). Expression of Fas (CD95/APO-1) antigen induced by radiation therapy for diffuse B-cell lymphoma: Immunohistochemical study. Clin. Cancer Res..

[8] Izawa S., Kono K., Mimura K., Kawaguchi Y., Watanabe M., Maruyama T., Fujii H. (2011). H_2_O_2_ production within tumor microenvironment inversely correlated with infiltration of CD56^dim^ NK cells in gastric and esophageal cancer: Possible mechanism of NK cell dysfunction. Cancer Immunol. Immunother..

[9] Martinez-Outschoorn U.E., Lin Z., Trimmer C., Flomenberg N., Wang C., Pavlides S., Pestell R.G., Howell A., Sotgia F., Lisanti M.P. (2011). Cancer cells metabolically “fertilize” the tumor microenvironment with hydrogen peroxide, driving the Warburg effect: Implications for PET imaging of human tumors. Cell Cycle.

[10] Ogawa Y., Nishioka A., Inomata T., Yoshida S., Nakayama K., Kataoka S. (1998). Radiation kills human peripheral T cells by a Fas-independent mechanism. Int. J. Mol. Med..

[11] Ogawa Y., Nishioka A., Kobayashi T., Kariya S., Hamasato S., Saibara T., Nakayama K., Seguchi H., Yoshida S. (2002). Mitochondrial cytochrome c release in radiation-induced apoptosis of human peripheral T cells. Int. J. Mol. Med..

[12] Ogawa Y., Nishioka A., Kobayashi T., Kariya S., Hamasato S., Saibara T., Seguchi H., Yoshida S. (2001). Radiation-induced apoptosis of human peripheral T cells: Analyses with c DNA expression arrays and mitochondrial membrane potential assay. Int. J. Mol. Med..

[13] Ogawa Y., Kobayashi T., Nishioka A., Kariya S., Hamasato S., Seguchi H., Yoshida S. (2003). Radiation-induced oxidative DNA damage, 8-oxoguanine, in human peripheral T cells. Int. J. Mol. Med..

[14] Ogawa Y., Kobayashi T., Nishioka A., Kariya S., Hamasato S., Seguchi H., Yoshida S. (2003). Radiation-induced reactive oxygen species (ROS) formation prior to oxidative DNA damage in human peripheral T cells. Int. J. Mol. Med..

[15] Ogawa Y., Kobayashi T., Nishioka A., Kariya S., Ohnishi T., Hamasato S., Seguchi H., Yoshida S. (2004). Reactive oxygen species-producing site in radiation-induced apoptosis of human peripheral T cells: Involvement of lysosomal membrane destabilization. Int. J. Mol. Med..

[16] Ogawa Y., Takahashi T., Kobayashi T., Kariya S., Nishioka A., Hamasato S., Moriki T., Seguchi H., Yoshida S., Sonobe H. (2004). Immunocytochemical characteristics of human osteosarcoma cell line HS-Os-1: Possible implication in apoptotic resistance against irradiation. Int. J. Mol. Med..

[17] Ogawa Y., Takahashi T., Kobayashi T., Kariya S., Nishioka A., Ohnishi T., Saibara T., Hamasato S., Tani T., Seguchi H. (2003). Apoptotic resistance of the human osteosarcoma cell line HS-Os-1 to irradiation is converted to apoptotic-susceptibility by hydrogen peroxide: A potent role of hydrogen peroxide as a new radiosensitizer. Int. J. Mol. Med..

[18] Ogawa Y., Takahashi T., Kobayashi T., Kariya S., Nishioka A., Mizobuchi H., Noguchi M., Hamasato S., Tani T., Seguchi H. (2003). Mechanism of apoptotic resistance of human osteosarcoma cell line, HS-Os-1, against irradiation. Int. J. Mol. Med..

[19] Kariya S., Sawada K., Kobayashi T., Karashima T., Shuin T., Nishioka A., Ogawa Y. (2009). Combination treatment of hydrogen peroxide and X-rays induces apoptosis in human prostate cancer PC-3 cells. Int. J. Radiat. Oncol. Biol. Phys..

[20] Ogawa Y., Takahashi T., Kobayashi T., Toda M., Nishioka A., Kariya S., Seguchi H., Yamamoto H., Yoshida S. (2003). Comparison of radiation-induced reactive oxygen species formation in adult articular chondrocytes and that in human peripheral T cells: Possible implication in radiosensitivity. Int. J. Mol. Med..

[21] Ogawa Y., Ue H., Tsuzuki K., Tadokoro M., Miyatake K., Sasaki T., Yokota N., Hamada N., Kariya S., Hitomi J. (2008). New radiosensitization treatment (KORTUC I) using hydrogen peroxide solution soaked gauze bolus for unresectable and superficially exposed neoplasms. Oncol. Rep..

[22] Lichtenbaum R., de Souza A.A., Jafar J.J. (2006). Intratumoral hydrogen peroxide injection during meningioma resection. Neurosurgery.

[23] Ogawa Y., Kubota K., Ue H., Tsuzuki K., Tadokoro M., Miyatake K., Sasaki T., Yokota N., Hamada N., Kariya S. (2007). Development and clinical application of a new radiosensitizer containing hydrogen peroxide and hyaluronic acid sodium for topical tumor injection—A new enzyme-targeting radiosensitization treatment, KORTUC II (Kochi Oxydol-Radiation Therapy for Unresectable Carcinomas, Type II). Strahlenther. Onkol..

[24] Tokuhiro S., Ogawa Y., Tsuzuki K., Akima R., Ue H., Kariya S., Nishioka A. (2010). Development of a new enzyme-targeting radiosensitizer (KORTUC) containing hydrogen peroxide for intratumoral injection for patients with low linear energy transfer (LET) radioresistant neoplasms. Oncol. Lett..

[25] Ogawa Y., Kubota K., Ue H., Kataoka Y., Tadokoro M., Miyatake K., Tsuzuki K., Yamanishi T., Itoh S., Hitomi J. (2009). Phase I study of a new radiosensitizer containing hydrogen peroxide and sodium hyaluronate for topical tumor injection: A new enzyme-targeting radiosensitization treatment, Kochi Oxydol-Radiation Therapy for Unresectable Carcinomas, Type II (KORTUC II). Int. J. Oncol..

[26] Prada P.J., Fernandez J., Martinez A.A., de la Rua A., Gonzalez J.M., Fernandez J.M., Juan G. (2007). Transperineal injection of hyaluronic acid in anterior perirectal fat to decrease rectal toxicity from radiation delivered with intensity-modulated brachytherapy or EBRT for prostate cancer patients. Int. J. Radiat. Oncol. Biol. Phys..

[27] Ogawa Y., Kubota K., Ue H., Tadokoro M., Matsui R., Yamanishi T., Hamada N., Kariya S., Nishioka A., Nakajima H. (2011). Safety and effectiveness of a new enzyme-targeting radiosensitization treatment (KORTUC II) for intratumoral injection for low-LET radioresistant tumors. Int. J. Oncol..

[28] Chasin W.D., Gross C.C., Wang C.C. (1967). Hydrogen peroxide and irradiation of tumors. Arch. Otolaryngol..

[29] Tsuzuki A., Ogawa Y., Kubota K., Tokuhiro S., Akima R., Yaogawa S., Itoh K., Yamada Y., Sasaki T., Onogawa M. (2011). Evaluation of changes in tumor shadows and microcalcifications on mammography following KORTUC II, a new radiosensitization treatment without any surgical procedure for elderly patients with stage I and II breast cancer. Cancers.

[30] Hitomi J., Kubota K., Ogawa Y., Hamada N., Murata Y., Nishioka A. (2010). Non-surgical therapy and radiologic assessment of stage I breast cancer treatment with novel enzyme-targeting radiosensitization: Kochi Oxydol-Radiation Therapy for unresectable carcinomas. Exp. Ther. Med..

[31] Ogawa Y., Kubota K., Aoyama N., Yamanishi T., Kariya S., Hamada N., Nogami M., Nishioka A., Onogawa M., Miyamura M. (2015). Non-surgical breast-conserving treatment (KORTUC-BCT) using a new radiosensitization method (KORTUC II) for patients with stage I or II breast cancer. Cancers.

[32] Yaogawa S., Ogawa Y., Morita-Tokuhiro S., Tsuzuki A., Akima R., Itoh K., Morio K., Yasunami H., Onogawa M., Kariya S. (2016). Serial assessment of therapeutic response to a new radiosensitization treatment, Kochi Oxydol-Radiation Therapy for Unresectable Carcinomas, Type II (KORTUC II), in patients with stage I/II breast cancer using breast contrast-enhanced magnetic resonance imaging. Cancers.

[33] Miyatake K., Kubota K., Ogawa Y., Hamada N., Murata Y., Nishioka A. (2010). Non-surgical care for locally advanced breast cancer: Radiologically assessed therapeutic outcome of a new enzyme-targeting radiosensitization treatment, Kochi Oxydol-Radiation Therapy for Unresectable Carcinomas, Type II (KORTUC II) with systemic chemotherapy. Oncol. Rep..

[34] Nishioka A., Ogawa Y., Mityatake K., Tadokoro M., Nogami M., Hamada N., Kubota K., Kariya S., Kohsaki T., Saibara T. (2014). Safety and efficacy of image-guided enzyme-targeting radiosensitization and intraoperative radiotherapy for locally advanced unresectable pancreatic cancer. Oncol Lett..

[35] Morita-Tokuhiro S., Ogawa Y., Yokota N., Tsuzuki A., Oda H., Ishida N., Aoyama N., Nishioka A. (2016). Development of a novel enzyme-targeting radiosensitizer (New KORTUC) using a gelatin-based hydrogel instead of a sodium hyaluronate. Cancers.

